# A TDD Framework for Automated Monitoring in Internet of Things with Machine Learning †

**DOI:** 10.3390/s22239498

**Published:** 2022-12-05

**Authors:** Victor Takashi Hayashi, Wilson Vicente Ruggiero, Júlio Cezar Estrella, Artino Quintino Filho, Matheus Ancelmo Pita, Reginaldo Arakaki, Cairo Ribeiro, Bruno Trazzi, Romeo Bulla

**Affiliations:** 1 Polytechnic School (EPUSP), University of São Paulo, São Paulo 05508-010, Brazil; 2 Institute of Mathematics and Computer Sciences (ICMC), University of São Paulo, São Paulo 13566-590, Brazil; 3 Electrical Engineering Deapartment, Federal University of Amapá (Unifap), Macapa 68903-436, Brazil; 4 School of Arts, Sciences and Humanities (EACH), University of São Paulo, São Paulo 03828-000, Brazil

**Keywords:** IoT, machine learning, software engineering, TDD, testbed

## Abstract

Robust, fault tolerant, and available systems are fundamental for the adoption of Internet of Things (IoT) in critical domains, such as finance, health, and safety. The IoT infrastructure is often used to collect a large amount of data to meet the business demands of Smart Cities, Industry 4.0, and Smart Home, but there is a opportunity to use these data to intrinsically monitor an IoT system in an autonomous way. A Test Driven Development (TDD) approach for automatic module assessment for ESP32 and ESP8266 IoT development devices based on unsupervised Machine Learning (ML) is proposed to monitor IoT device status. A framework consisting of business drivers, non-functional requirements, engineering view, dynamic system evaluation, and recommendations phases is proposed to be used with the TDD development tool. The proposal is evaluated in academic and smart home study cases with 25 devices, consisting of 15 different firmware versions collected in one week, with a total of over 550,000 IoT status readings. The K-Means algorithm was applied to free memory available, internal temperature, and Wi-Fi level metrics to automatically monitor the IoT devices under development to identify device constraints violation and provide insights for monitoring frequency configuration of different firmware versions. To the best of the authors’ knowledge, it is the first TDD approach for IoT module automatic assessment which uses machine learning based on the real testbed data. The IoT status monitoring and the Python scripts for model training and inference with K-Means algorithm are available under a Creative Commons license.

## 1. Introduction

The Internet of Things (IoT) is a critical infrastructure necessary to the deployment of large projects with huge potential to change whole business sectors. Things present in IoT environments are responsible for measuring the variables of the physical world and share it across the network. Either a local network or the Internet can be used to connect things and generate a connected environment that can be measured and controlled. Sensors embedded in these things enable the measurement of temperature, pressure, oxygen levels, presence, and others that are applied to projects related to Smart Cities, Industry 4.0, and Big Data.

Consider an example of IoT application in the remote operation field. Workers operating remotely on hazardous situations and dangerous scenarios, with reduced costs and safety guaranteed by the distance and the real time monitoring [1]. IoT could be the bridge between the IT and the operations ground using the data generated in the production process [2]. The non-functional requirements of the underlying IoT infrastructure are fundamental to enable the business value generation in this example.

Moreover, critical applications, such as health, finance, and safety, require higher levels of quality. These areas are good examples of critical areas that require robust and fault tolerant systems with high availability, as they are used in fields that are crucial for the function of society, and unavailability events in these areas can be dangerous and expensive. As analyzed by IBM in 1998 [3], the unavailability of brokerage operations and credit cards operations had an average combined cost of USD 9.1 million per hour.

Considering an IoT architecture organized in layers as described in the reference architecture IoT-A [4], the applications, such as the Smart City, are present in the data layer, corresponding to the Application and Presentation layers of the traditional OSI model. The underlying layers, End to end, Network and ID, Link, and Physical, which correspond to the Session, Transport, Network, Data Link, and Physical layers of the OSI model. Similar to how the Internet works, the underlying layers are essential for the smooth operation of the applications, and we consider that these layers embed the non-functional requirements that shape quality levels necessary to critical applications. The successful deployment of IoT depends on how these non-functional requirements are modeled and implemented to provide a reliable and trustworthy infrastructure that enables the IoT to create value to businesses and people.

However, the deployment of traditional approaches to the IoT domain is not trivial. The IoT integrates constrained devices with energy, processing, memory, and communication limitations, and it also employs heterogeneous communication protocols, devices, and architectures, such as those that are edge and cloud-based. A motivation for this work is relative to the application of a similar protocol to the Simple Network Management Protocol (SNMP) [5] to manage IoT devices considering monitoring procedures.

In this article, we consider the following research question: “is it possible to assess if non-functional requirements are being supported by an IoT system in real time in an autonomous way?”. The objective is to monitor IoT modules with the goal of increasing its Testability. Testability is considered a sub-characteristic of the Maintainability non-functional characteristic as described in ISO25010 [6].

The problem considered within the scope of this paper is the lack of automated testing and monitoring of IoT devices under development. Such low Testability hinders the quality aspects of the resulting IoT system. For example, if an IoT device violates its resource utilization in the development phase, the resulting IoT product may present unavailability events when deployed.

Our hypothesis considers that the RM-ODP [7] standard is a relevant reference for the proposition of a Test-Driven Development (TDD) Framework to allow the monitoring of IoT systems considering a structured approach considering business drivers, non-functional requirements, and dynamic evaluation. The TDD [8] is applied with unsupervised machine learning based on IoT deployed modules data to obtain automated tests that better represent real-world constraints. These automated tests could be used by a developer with a TDD approach, so that IoT module problems (e.g., memory limitation) are identified earlier in the development process.

The main contributions of this work are:

- A Test-Driven Development (TDD) tool built with K-Means clustering algorithm with real testbed data to enhance the Testability non-functional requirement of IoT systems under development;
- A framework that considers business drivers and non-functional requirements to support the IoT solution development process;
- Validation of a proposed framework in smart home and remote lab study cases to obtain deployment conditions and monitoring frequency of different IoT device firmware versions.

This article is an extended version of our paper published in Intercloud and IoT at 8th International Conference on Future Internet of Things and Cloud (FiCloud 2021), considering the historical data collection, the addition of the remote lab case study, and the proposition of a generalized framework considering RM-ODP besides the TDD.

To the best of the authors’ knowledge, it is the first TDD approach for IoT module automatic assessment which uses machine learning based on real testbed data. Some of the related work carried out experiments in testbeds with real hardware devices, but none of them used machine learning, which motivated the novel TDD approach validated with two study cases.

The open source materials related to this article are available in the following repository: https://github.com/vthayashi/TDD-IoT, (accessed on 30 November 2022).

This document is organized as follows: Section 2 presents a structured literature search and with some related work. Section 3 follows with the Research Background of IoT architecture, ISO25010 as a standard for non-functional requirements, the RM-ODP standard, the microservices architecture and the TDD. Our proposed framework is presented in Section 4. Section 5 describes the smart home and remote lab study cases to show how the framework might be applied in the wild to elaborate suggestions related to microservices, cloud and edge architectures, and device management. We discuss and compare our approach with the related work in the Section 6. The final considerations and directions for related work are presented in Section 7.

## 2. Related Work

The related work [9] offers an overview of the technology requirements for future Cooperative Cyber–Physical Systems (Co-CPSs) in terms of wireless communication and safety.They focused on highlighting the main wireless communication technologies currently available and evaluated their compliance to the safety requirements of Cooperative Cyber–Physical Systems (Co-CPSs) in terms of dependability, security and safety.

The researchers in [10] presented a novel hybrid-simulation-based testing technique. This technique allows IoT systems to be tested by orchestrating a real-time interaction between real-life and virtual local IoT entities. First, they analyze the behavior of local entities, such as IoT devices or people interacting with the IoT system. Additionally, they presented two possible techniques to solve the scalability constraints of state-of-the-art simulation techniques: one based on model abstraction and another based on the optimal resource distribution of simulation entities. Challenges that arise when implementing this hybrid simulation based technique are the simulation of the human behavior and the lack of synchronization.

Developers in [11] demonstrated how an IoT-based infrastructure could support establishing a co-simulation platform following SGAM requirements. Integrating a digital real-time simulator could enable to test physical devices by hardware in-the-loop set-ups, as well as evaluating the performance of new algorithms via software in-the-loop experiments. The results of the proposed architecture are not as precise as a real advanced measurement infrastructure, but it could provide accurate results.

Researchers in [12] compare open source technologies of IoT from the point of view of different levels of technical requirements, such as device management, data management, communication, data processing, security and privacy protection, and also look at requirements of application development and deployment. Then, a cloud–fog cooperated integrated application development platform architecture for IoT applications based on an open source ecosystem was proposed and evaluated in an industrial IoT scenario. The performance results showed that the CoAP protocol performed better than HTTP. The gateway availability test also showed that the IoT gateway based on the open source ecosystem had a stable and reliable performance with a certain data size and concurrency scale, and that this scale could meet the application requirements of the IoT in most sensing environments.

Researchers in [13] proposed a layered IoT architecture (called IoTecture) whose components are mapped to stages of an IoT computing in different deployment configurations. A performance analysis study with six configurations revealed that different deployment configurations of layered components into staged locations generate different hardware and software bottlenecks that affect system performance and scalability. Examples of limitations of this article are: evaluating the performance of components located in higher layers; automating the deployment process; and using different technology sets.

The researchers in [14] proposed an integrated authentication and capability-based access control for increased usability in IoT environments. The important characteristic of the approach is that the capability metric generated during authentication is used to perform access control. The approach also allows lightweight operations to be performed on IoT devices and computation intensive operations on the cloud server. The security evaluation also shows that it is secure against various attack vectors predominant in IoT. The testbed used a laptop device to imitate the Cloud Server and Raspberry Pi devices to imitate the IoT devices. The experimental results show that the proposed approach incurs a maximum CPU usage of 29.35%, a maximum memory usage of 2.79%, and total computational overhead of 809.26 ms in a real IoT testbed.

In [15], a routing protocol for the IoT (RIoT) was presented with an aim to overcome the problems associated with routing protocol for low-power and lossy networks (RPL) for a better IoT ecosystem. They also presented a communication architecture based on multiple gateways. The testbed results demonstrate that due to RIoT’s lower control message overhead it exhibits better performance in terms of packet delivery ratio (PDR) and per-packet end-to-end delay compared to the RPL-based protocol. They showed that network partitioning is a major problem in mobility-based IoT use cases, and the impact of the problem on a network performance can be reduced using multiple gateways in the network.

The proposal in [16] is to use the MAPE-K framework (monitoring, analyze, plan, and execute with knowledge base), applied to a BPM system to operate an IOT environment capable of monitoring itself in cycles and verifying its own functioning and its own quality, if the tasks are being carried out in the best way possible.

Researchers in [17] presented an alternative to improve the data transmission validation process in a previously developed WSN-IoT system. They implemented the test-driven development methodology (TDD) for the build out of validation application algorithms. As a result, they developed validation application that captures and analyzes data frames, in addition, compares the values of the monitored variable in each layer of the implemented model. Furthermore, the development reports the errors detected in the data transmission. As limitations of this work, they cite the high dependence on networks to monitor the performance of the designed application.

In [18], the researchers described the Open-Multinet (OMN) framework, which allows the extraction of underlying information from tree-based data structures. Additionally, they developed a set of ontologies to support resource management in federated and distributed computing infrastructures. Users can query OMN information that represents the resources available in the underlying infrastructures and match them with their own computational requirements. The time needed to find matching resources in more complex queries are acceptable to end-users.

A mathematical model to represent a system in aggressive cyberspace is presented in [19], and it supported a simulation of a system considering its efficiency of operation, operation risk, and the resources invested in cybersecurity measures.

The authors of [20] propose the functional safety estimate of a cyber–physical system using a Markov model. It is relevant because the assessment of functional safety is one of the primary tasks both at the design stage and at the stage of operation of critical infrastructure, in which IoT systems could be deployed.

Regarding IoT systems development, some application fields are vehicular networks (e.g., delay-tolerant networks for IoT-based vehicular networks are proposed in [21]) and smart homes (e.g., a smart home based on Bluetooth is proposed in [22]). One case study presented in this paper is also in the smart home scenario.

Table 1 summarizes the related work. We realized that some of the related work carried out experiments in testbeds with real hardware devices but none of them used machine learning (ML), which motivates the TDD approach with machine learning proposed and validated with two study cases.

## 3. Research Background

This section presents the IoT architecture considered in this work, the ISO25010 standard to understand the non-functional requirements used to specify quality levels the resulting IoT system must adhere. The RM-ODP method is described as a structured approach to consider different views during a system specification and modeling. The microservices architecture is described considering its strong and weak points. Finally, the TDD methodology is summarized as a guideline we used in the proposition of the automated monitoring based on automated tests.

### 3.1. IoT Architecture

Standards and reference architectures (RA) contribute to the reduction of the complexity of IoT systems and the architectural structures help to build IoT systems that can support and execute necessary functions [23]. It is important because an architecture for IoT needs to integrate many technologies, such as protocols, patterns, sensors, actuators, conventional frameworks of the Internet, and applications for the most different purposes while keeping the security and reliability of applications.

IoT architecture presents challenges in the most diverse scenarios. These challenges may be in architectural layers (inter-architecture or intra-architecture), entity-based (hardware, software, or data), technological (specific IoT or related technologies), or feature-based challenges (compatibility, capacity, connectivity, scalability, or security) [24]. Then, the challenges for architecture in IoT are diverse and occur because of the heterogeneity of environments and technologies.

IoT heterogeneity can be observed in [25] with an overview of IoT architectures, taxonomies, devices, gateways, operating systems, communication technologies, middleware, and platforms. Architectures based on layers, middleware, service-oriented, and fog-based are presented. In middleware-based architectures, one middleware layer is responsible for controlling the flow of data in the system. Service-oriented architecture divides the functionalities and exposes them through interfaces. In fog-oriented architectures, other layers are added with a focus on the processing of data.

Therefore, the diversity of IoT architectures result in its complexity. Reference architectures are proposed to mitigate this diversity. Another issue must be taken into account, the heterogeneity of physical and software components to be integrated in these architectures. These components directly influence the complexity of the IoT design and the choice of architecture.

In the proposed TDD framework, we propose the automated monitoring of black-box connected devices and software modules that compose IoT architectures. As long as the architecture uses the open standard MQTT (Message Queuing Telemetry Transport) [26] protocol to support rastreability with the proposed framework, the underlying IoT architecture may use other technologies and reference architectures. In this way, the proposed framework may contribute to address the challenge of diverse IoT architectures by enhancing their testability.

### 3.2. ISO25010

There are development guidelines to be followed in order to ensure that the IoT system delivers the needed level of trust and reliability, with non-functional requirements to be fulfilled. By definition, the non-functional requirements of a system are what the system is, not what the system does. The non-functional requirements must be evaluated under a quality model that assures the quality in a degree that the system can satisfy the needs and demands of its stakeholders. The ISO/IEC model defines the standard of non-functional requirements and guarantees that each of the stakeholders needs are covered.

The ISO25010 standard defines a quality model composed of eight quality characteristics: functional suitability, performance efficiency, compatibility, usability, reliability, security, maintainability, and portability. The performance efficiency considers the time behavior, resource utilization and capacity of a system. The maintainability characteristic has the modularity, reusability, analysability, modifiability, and testability characteristics.

The development process must be planned in order to be compliant with the ISO/IEC 25010 [6] from the beginning, combining characteristics with agile development methods to deliver fault tolerant, reliable, and trustworthy software solutions. As an example of the implementation of a software engineering tactic to control a non-functional requirement of an IoT system, consider the Markov analysis for availability evaluation and impact of preventive and corrective maintenance procedures at device and local network levels based on real testbed data from local and Internet-level ping procedures [27].

The proposed framework aims to enhance the testability, which is a characteristic of the maintainability non-functional requirement of ISO25010 standard. The study cases reported in the next sections investigated the automated monitoring of the resource utilization, characteristic of the performance efficiency non-functional requirement.

### 3.3. RM-ODP

The RM-ODP method [7] provides five architectural viewpoints that complement each other: business domain (consisting of business drivers that the solution must comply), functional and non-functional requirements, engineering solutions (considering hardware, software, and process mechanisms), and technologies (components, platforms, and their integrations). It provides a rationale to specify solutions from the business domain, to the requirements, engineering solution and technologies that are used to implement the system.

Considering the trade-off involving high capacity in the IoT scenario in terms of processing from cloud resources, and limited capacity of vulnerable sensors and actuators, these views from RM-ODP contribute to identify architectural decisions that must be carefully created, tested, and monitored through all the development and operation life cycle to support quality aspects, such as fault tolerance, availability, precision, and traceability.

Simulations regarding trade-off analysis in IoT architectures are presented in the literature. For example, the authors of [28] use Petri Nets to evaluate architectural trade-offs in a smart speaker system, such as the accuracy and response time trade-off between speech to text services, and provided some suggestions regarding a redundant architecture and the lock-in aspect of some conversational interfaces integrated to IoT systems.

The case studies presented in the following sections use the RM-ODP views to organize the documentation of the automated monitoring tests.

### 3.4. Microservices

A monolith structure means high coupling in terms of data, functionality, and technology. Low development scale, low productivity in tests and limited evolution are some characteristics in opposition with an agile development process. There are non-functional requirements as the foundation to adopt microservices [29,30] structures for IoT systems.

Sensors precision, availability, and resulting data integrity considering vulnerable devices. In this scenario, fault tolerance is achieved by microservices which deal with the low robustness of devices, so that the fragility of specific parts of the system do not damage the rest of the IoT application (if well isolated).

The fault tolerance aspect needs redundancies of hardware, software, and data algorithms with high cohesion and low coupling structuring components. Small parts command alternative functions that are sufficiently complete to avoid system unavailability because of these smart redundancies provided by independent services.

Modifiability would be one of the leading quality aspects of successful microservices adoption. The modification and test spaces are limited to pieces that bring agility, velocity for changes, and business evolution due to the flexible architectural solution.

An IoT system requires a balanced architecture to support agility to evolve, perform, monitor, and respond considering suitable functions and the large scale of a parallel development team. All of these aspects are present in the microservices-based architecture. Additional benefits are to be achieved if the agile development process, automation tests, and continuous integration are combined with the microservices architecture.

### 3.5. TDD

The manifest of agile software development main point is to focus more on what makes the solution work than in rules and writing about the solution [31]. The goal is to deliver the best of the solutions, with continuous improvement during the development process. For testing, Kent Beck, signer of the agile manifest, adds Test-Driven Development (TDD) to the manifest concepts and states that test cases should be written for the needed improvement, and then code should be written for this test and refined until it becomes acceptable [8].

The TDD seems easy to write but difficult to execute. In a microservices-based system, the TDD is so crucial as it is related to properly testing components (code and assets) of the IoT system. If an automation test module is responsible for approving modified parts, then an automated quality gate is working. Additional software process challenges arise with non-functional requirements; response time, precision, simultaneous access, and fault-tolerance are examples of quality aspects that might require automated verification:

- Test programs may create conditions with high simultaneous access then measure the response time to assess good performance levels;
- Similar to precision, using simulated fault of sensor readings to validate the employed redundancies to support precise responses;
- In case of simultaneous access, the first item would be sufficient to create a quality gate to approve modifications;
- Availability can be measured by test cases that create fault parts of the system and the resultant behaviors.

As the development process follows the test driven methodology, an opportunity arises: the technology itself could be used to ensure the system compliance levels. In this work, we consider the automation of the test cases based on data collected by deployed modules ensures that these tests are close enough to the real-world scenario.

## 4. Proposed Framework

This section presents the proposed framework for IoT automated monitoring. First, the proposed method based on TDD and RM-ODP is described. The automated test approach detailing how the unsupervised machine learning is used on real testbed data follows. Finally, an IoT status example for the ESP32 IoT Development Module is presented.

### 4.1. Proposed Method

**Figure 1:**
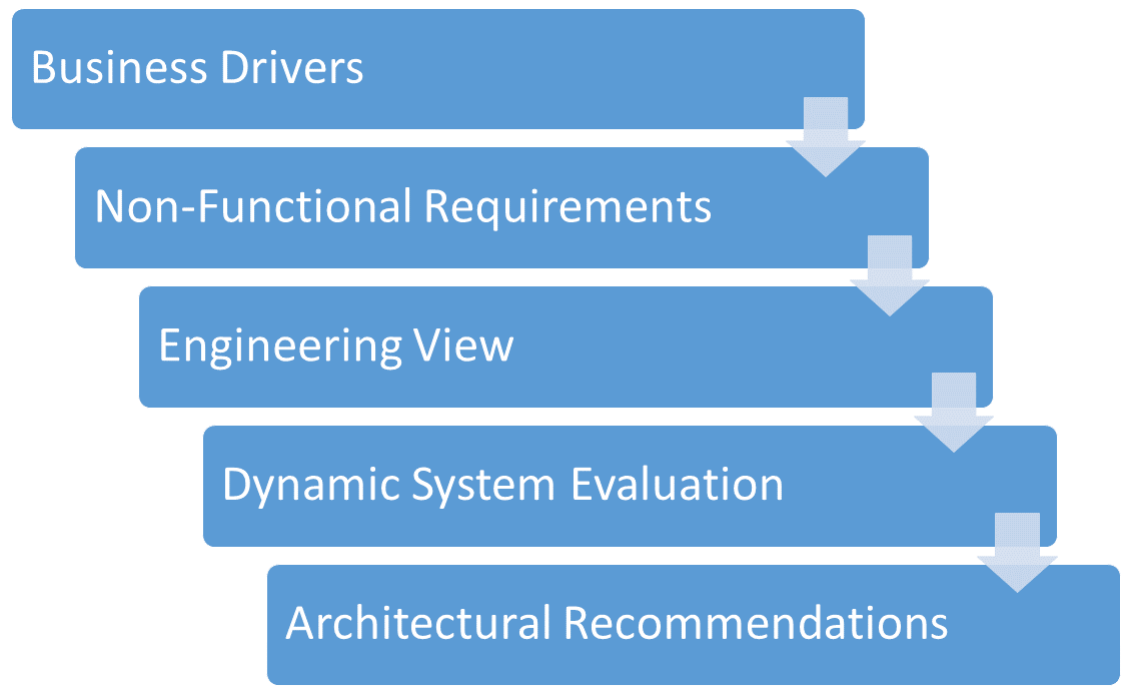
Proposed method for the framework for automated monitoring in IoT.

The proposed method considered in the framework for automated monitoring in IoT is illustrated in the Figure 1. It must be used by a team consisting of different stakeholder roles:

- IoT developer: the technical staff, individual, or company that is held responsible by the implementation of the IoT solution. It must have necessary hardware, software and architectural expertise to develop and deploy the IoT solution;
- Business specialist: usually the client, a team, or individual that wants to deploy IoT solutions to reduce costs and create value in Smart City, Industry 4.0, Smart Home, and other application fields. It has a deep understanding of the business needs related to the IoT solution.

We consider that the IoT developer and the business specialist are individuals or teams that participate in an IoT project in a critical field. The quality aspects must be controlled to make sure that the resulting technical solution adheres to the non-functional requirements modeled considering the business drivers. The method may be applied in the start of an IoT project to enhance the resulting IoT solution quality attributes. It is comprised of the following steps:

1. Business drivers: related to business view from the RM-ODP. Business needs must be specified by the business specialist to the IoT developer;
2. Non-functional requirements: according to the ISO25010, specify the non-functional requirements and prioritize these requirements considering the business drivers described in the first step;
3. Engineering view: related to the engineering view present in RM-ODP. The IoT developer must present the architectural solution that support the non-functional requirements monitoring;
4. Dynamic system evaluation: real time monitoring and data collection from testbeds that enable automated tests and analysis with unsupervised machine learning mechanisms. The automated tests are used by the IoT developer in a TDD methodology;
5. Recommendations: the IoT developer must provide suggestions based on the analysis performed to the business specialist, considering how the business drivers are modeled in non-functional requirements. The suggestions may be related to the architecture (e.g., monolithic or microservices, cloud or edge data processing).

### 4.2. Automated Test

**Figure 2:**
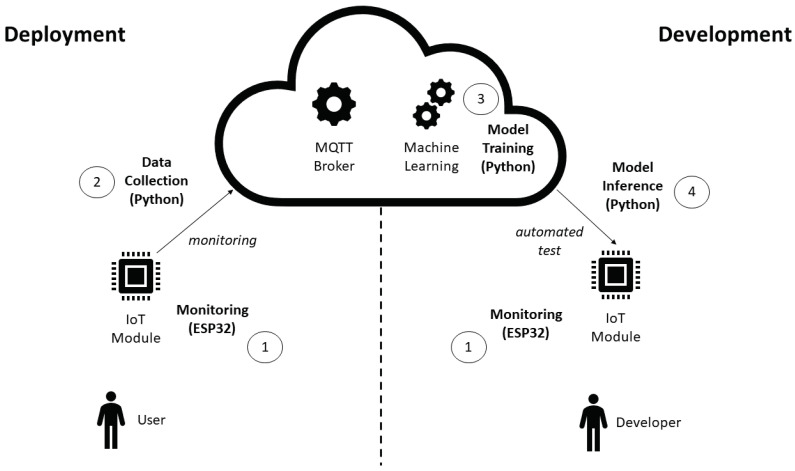
Proposed TDD scheme for IoT with automated tests.

The proposed automated test approach is illustrated in Figure 2. There are three environments considered: the deployment, cloud, and development environment. The user interacts with IoT modules in an deployment environment, such as a smart home. The deployed modules send their status to a MQTT (Message Queuing Telemetry Transport) [26] broker in a continuous fashion. The MQTT broker available in a cloud computing environment, where other services such as storage could also be available. The IoT module status metrics are used to train an unsupervised machine learning model. This trained model is used in an automated test that the developer applies to an IoT module under development. As the model was trained with the deployment scenario data, we advocate that the resulting automated test resemblance to a real-world scenario is bigger than a manual test that the developer could create.

**Figure 3:**
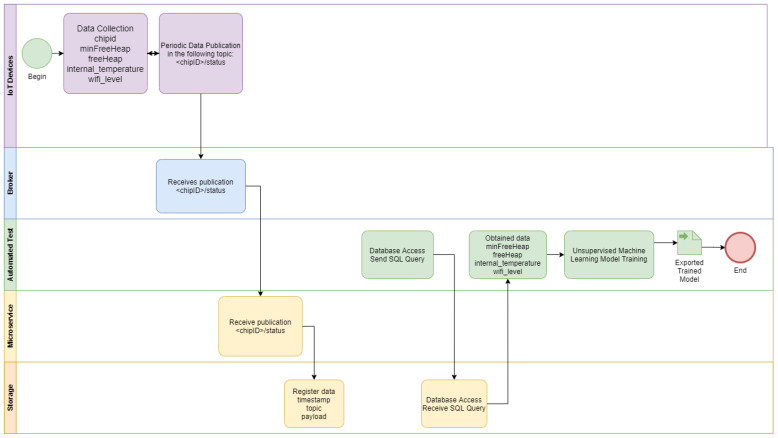
BPMN diagram of the training phase of the automated tests.

**Figure 4:**
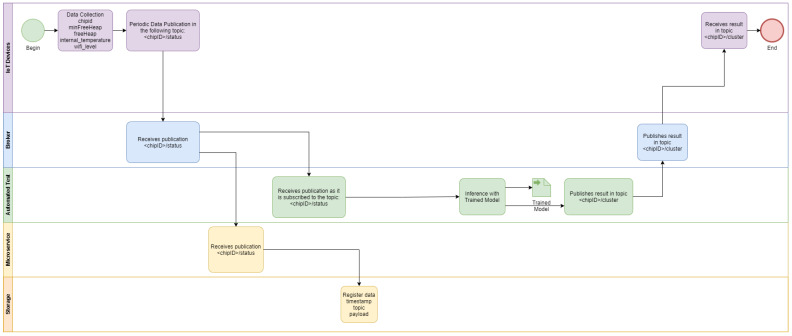
BPMN diagram of the inference phase of the automated tests.

ESP32 modules with different firmware versions (and even for different applications) send their data frequently to an open MQTT broker using the integrated Wi-Fi communication (1). A script is responsible for data collection from the open MQTT broker deployed in a cloud computing environment (2). The data collected are used to train the K-Means model with another script deployed in Google Colab cloud environment, with the cluster labeling (i.e., good or bad) performed by the developer (3), as detailed in Figure 3. The trained model is imported by a script responsible for inference that receives the real time data from the ESP32 modules and classify them according to the labels provided by the developer in the previous step (4). The inference procedures is detailed in the BPMN diagram of Figure 4. The K-Means clustering algorithm [32] was chosen because of its maturity. An additional motivation is that this algorithm showed good results in other applications, such as image segmentation [33].

### 4.3. IoT Status

**Figure 5:**
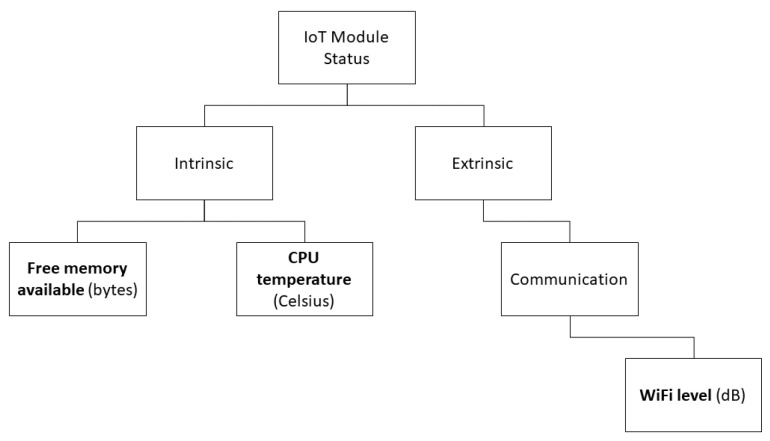
Example of IoT module status collected metrics.

As illustrated in Figure 5, the IoT module status is composed by intrinsic and extrinsic metrics. Intrinsic metrics could be the total free memory available in a given moment, or the CPU temperature. An example of extrinsic metric is related to communication, that is subject to external environmental conditions. In this example, we consider the ESP32 which has a built-in Wi-Fi communication, it is possible to obtain the Wi-Fi level of the network which the IoT module is connected to.

**Figure 6:**
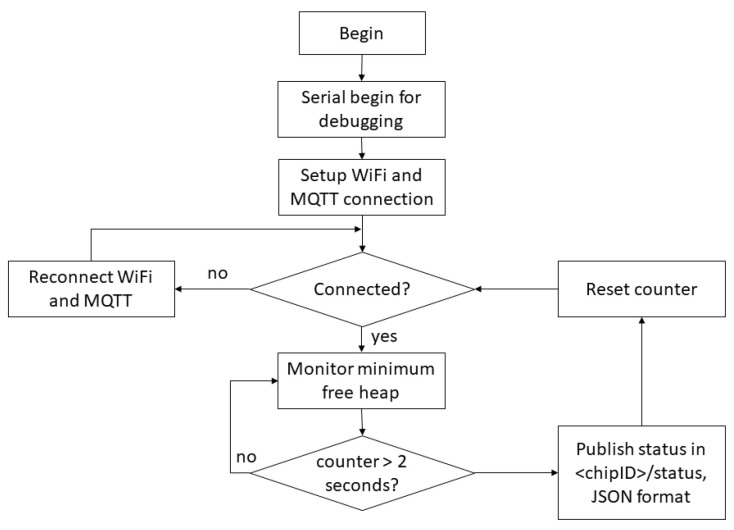
IoT module flowchart for status publishing in a MQTT topic.

The metrics free memory available, processor’s temperature and Wi-Fi level may be collected with the ESP32. Figure 6 portrays the IoT status monitoring rationale to collect these three metrics. Wi-Fi and MQTT connection are monitored and reconnection procedures are applied if necessary. If both connections are available, then the metrics monitored are sent to the MQTT broker in JSON format each 2 s. Each module publishes in a specific topic “chipID/status”, where “chipID” is an identifier available for the ESP32 module. As the free memory available is an instantaneous variable, it is monitored with the highest frequency possible, and its minimum value is stored and sent to the MQTT broker.

We validate our approach with a Proof of Concept (PoC) with ESP32 and ESP8266 (its predecessor) Wemos IoT Development modules. The libraries used for the IoT module monitoring script are Wi-FiClient and Wi-Fi for Wi-Fi connection, and PubSubClient for MQTT connection.

## 5. Case Studies

This section presents two study cases that show how the proposed method could be applied in the wild with different MQTT broker implementations, SQL and NoSQL databases, and in private and public cloud environments.

### 5.1. Remote Lab

**Figure 7:**
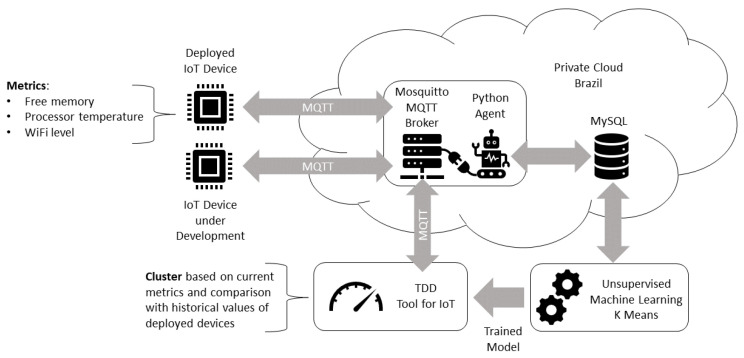
Remote lab study case architecture.

The first study case is a remote lab whose IoT devices are responsible for temperature and humidity monitoring, and air conditioner control by infra-red signals. As illustrated in the Figure 7, each deployed IoT device sends its status readings to a Mosquitto MQTT broker. The status available for the ESP32 devices in this scenario is composed by free memory, processor temperature and Wi-Fi level metrics. A Python agent is integrated with the MQTT broker and it is responsible for the registration of messages in a MySQL database. This database is consulted by a Python notebook for an unsupervised machine learning training. The trained K-Means model is used by a TDD tool that uses the current IoT status readings to assess if the IoT under development has similar status to the deployed IoT devices, according to their historical values. The Mosquitto MQTT broker, Python Agent, and MySQL database are deployed in a private cloud computing environment in Brazil, the deployed IoT device is in a remote lab in an Brazilian University, the IoT device under development is installed in a student’s home, where the TDD tool and model training Python notebooks are executed.

**Figure 8:**
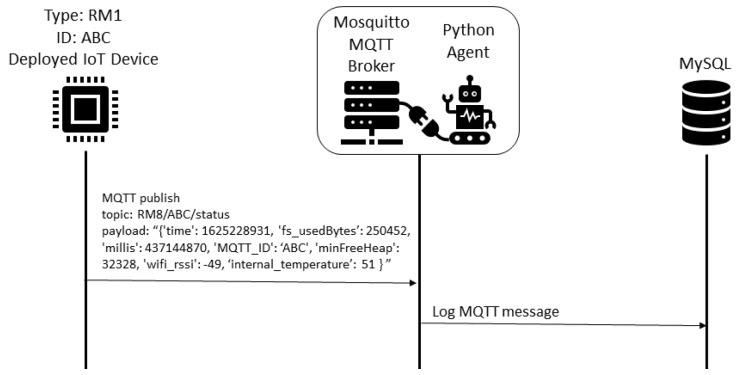
Deployed IoT device status data collection with periodic sending.

The data collection procedure is detailed in the Figure 8. Each 10 s the air conditioner devices send the status to the MQTT broker, and the humidity and temperature device send the status each 2 s. All messages are registered in the MySQL database by the Python Agent integrated with the Mosquitto MQTT broker.

**Figure 9:**
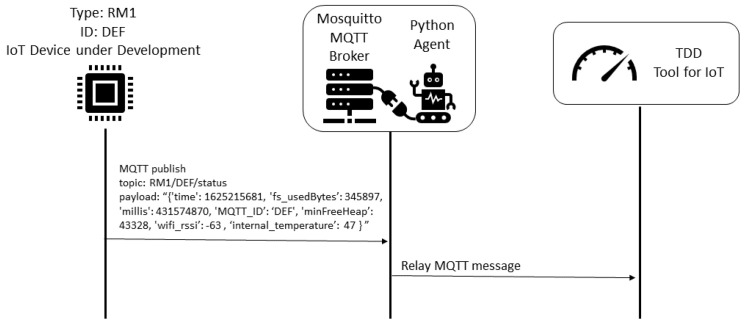
TDD procedure for IoT device under development (remote lab scenario).

The TDD procedure is described in the Figure 9. The TDD tool with the trained machine learning model is subscribed in IoT status topics. Each status message from the IoT device under development is relayed to the TDD tool by the MQTT broker, and it is used to compare the current status to the clustered historical values from deployed devices.

We collected a total of 1178 readings from two ESP32 devices with two different firmware versions from 9 July 2021 to 13 July 2021. The metrics of the ESP32 devices are freeheap in bytes (i.e., free memory available), internal_temperature in Celsius (i.e., temperature of the processor) and wifi_level in decibels (i.e., local Wi-Fi network level). A summary of the collected metrics of these two devices is presented in the Table 2.

**Figure 10:**
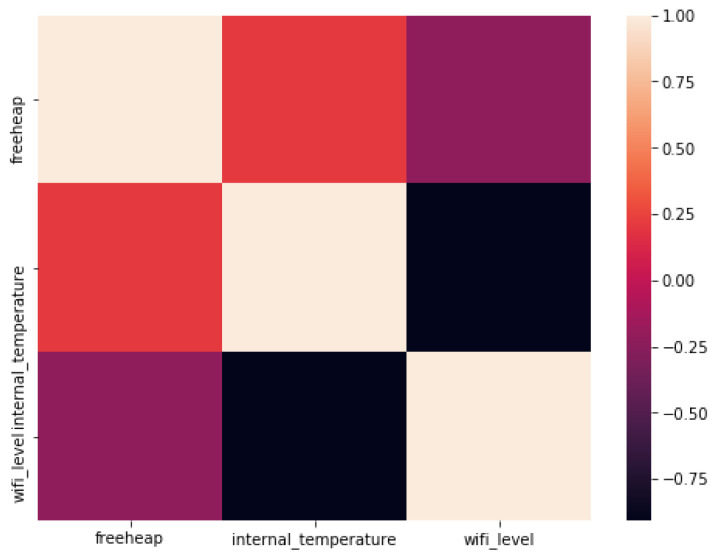
Remote lab metrics correlation matrix.

The correlation matrix of the Figure 10 shows the strong negative correlation between wifi_level and freeheap.

**Figure 11:**
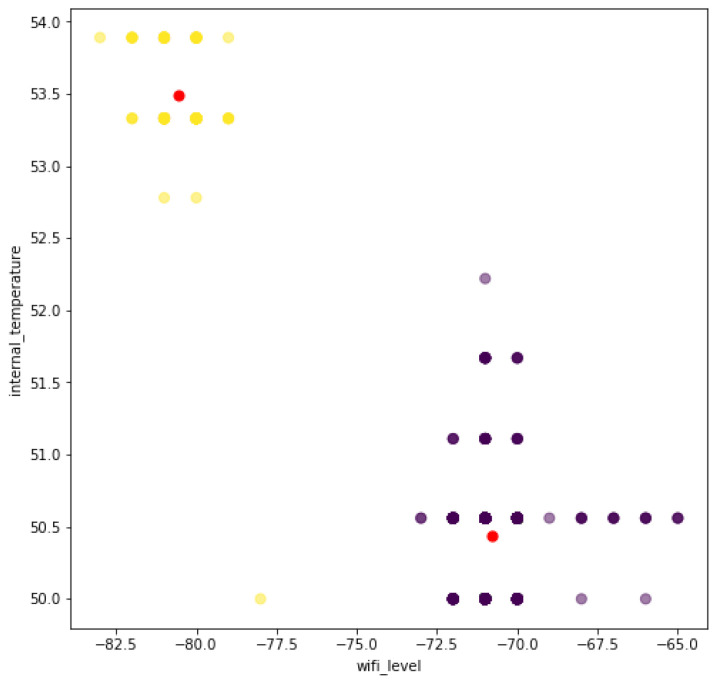
Remote lab K-Means clusters. Red dots are the centroids of each cluster, cluster 0 has yellow dots and cluster 1 has purple dots.

The negative correlation between wifi_level and freeheap is confirmed by the clusters visualization in Figure 11. The K-Means clustering algorithm was performed using the aforementioned three metrics with the total number of cluster of two corresponding to a good and bad status clusters. After the clustering, the cluster label is performed by the IoT developer based on the visual representation, such as the one represented in Figure 11.

The clustering results are summarized in Table 3. One may observe that both firmwares presented more bad status occurrences than good status occurrences, with bad status occurrences accounting for around 60% of all readings for both cases.

With the trained model, it was possible to classify the IoT device under development status described in (1) in the bad status cluster (i.e., cluster 0).

(1) $$\begin{array}{c} {}^{\prime }chipid^{\prime }{:}^{\prime }16716748^{\prime }\\ {}^{\prime }freeheap^{\prime }{:}^{\prime }222160^{\prime }\\ {}^{\prime }internal\_temperature^{\prime }{:}^{\prime }53.89^{\prime }\\ {}^{\prime }wifi\_level^{\prime }{:}^{\prime }-81^{\prime } \end{array}$$

Considering the proposed framework, we describe the five steps considered:

1. Business drivers: the motivation is to scale the air conditioner automation solution to the entire campus, considering the different communication conditions, and the possible additional functionalities that may be required by certain labs;
2. Non-functional requirements: the performance efficiency requirement is prioritized, considering the resource utilization. Another relevant requirement is the maintainability, considering the testability characteristic.
3. Engineering view: the architectural solution was illustrated in the Figure 7, and described in detail.
4. Dynamic system evaluation: initial descriptions are described in Figure 8 and Figure 9. The historical clustering results are presented in Table 3, and one example of a status reading classified in the cluster 0 (bad status according to the IoT developer rationale) is presented in (1).
5. Recommendations: considering that the deployed modules presented Wi-Fi levels from −83 to −65 (see Figure 11), it is desirable that the new IoT automation modules have Wi-Fi levels within this range. Based on Table 2, the modules with extended functionalities must maintain a free memory of around 220,000 bytes. The developers should use the TDD tool to monitor the free memory and internal temperature metrics.

### 5.2. Smart Home

The second study case is a smart home whose IoT devices are responsible for energy consumption monitoring, and appliances control by smart plugs.

**Figure 12:**
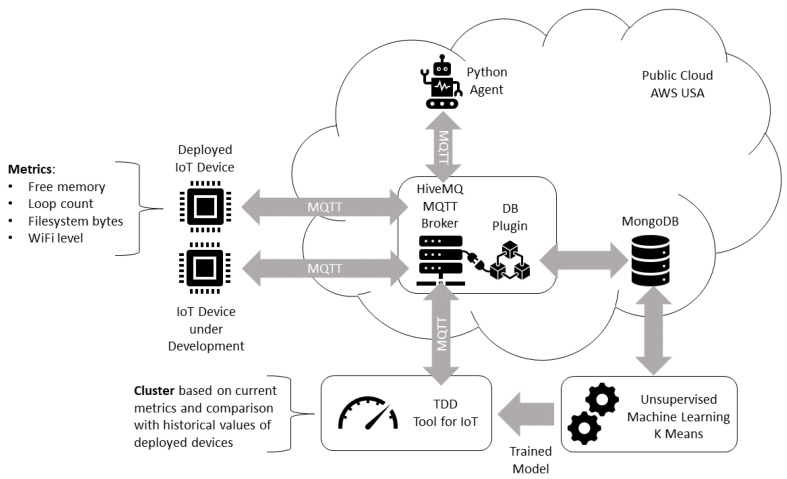
Smart Home study case architecture.

As illustrated in the Figure 12, a Python agent requests the IoT status in a periodic and configurable way. Each deployed IoT device responds by sending its status readings to a HiveMQ MQTT broker (Community Edition, which is the free version). The status available for the ESP8266 devices in this scenario is composed by free memory, loop count, total bytes used by the filesystem, and Wi-Fi level metrics. A database plug-in is integrated with the MQTT broker and it is responsible for the registration of messages in a MongoDB NoSQL database. This database is consulted by a Python notebook for an unsupervised machine learning training. The trained K-Means model is used by a TDD tool that uses the current IoT status readings to assess if the IoT under development has similar status to the deployed IoT devices in the smart home, according to their historical values. The HiveMQ MQTT broker and its database plug-in, Python Agent, and NoSQL database are deployed in a public cloud computing environment in the United States (USA), the deployed IoT device is in a Brazilian smart home testbed, and the IoT device under development is installed in a student’s home, where the TDD tool and model training Python notebooks are executed.

**Figure 13:**
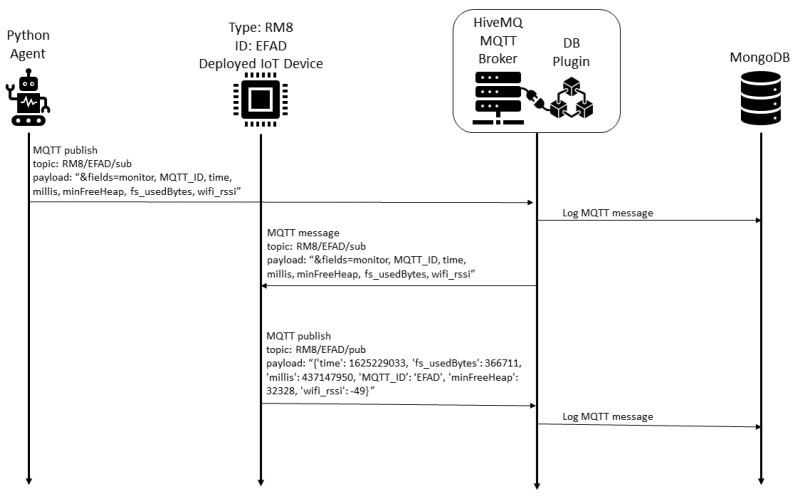
Deployed IoT device status data collection with periodic request.

The data collection procedure is detailed in the Figure 13. Each second the Python agent queries the IoT devices deployed in the smart home, which send their status to the MQTT broker. All messages are registered in the NoSQL database by the plug-in integrated with the HiveMQ MQTT broker.

**Figure 14:**
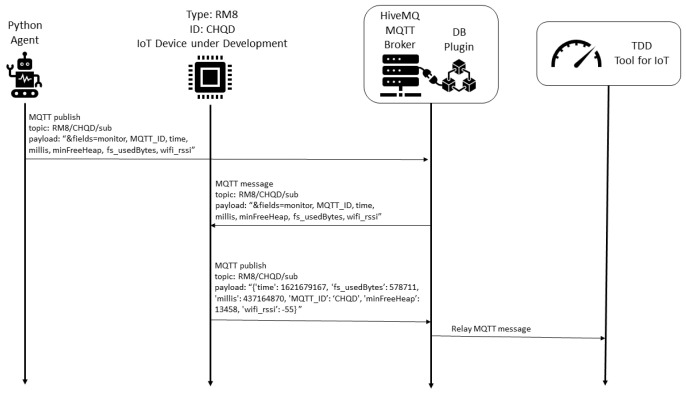
TDD procedure for IoT device under development (smart home scenario).

The TDD procedure is described in the Figure 14. The TDD tool with the trained machine learning model is subscribed in IoT status topics. Each status message from the IoT device under development (sent as a result of the periodic Python Agent request) is relayed to the TDD tool by the MQTT broker, and it is used to compare the current status to the clustered historical values from deployed devices.

We collected a total of 595,554 readings from 22 ESP8266 devices with 13 different firmware versions from 10 July 2021 to 17 July 2021. The metrics of the ESP8266 devices are freeheap in bytes (i.e., free memory available), wifi_level in decibels (i.e., local Wi-Fi network level), fs_usedbytes (i.e., total bytes used in the filesystem) and max_loop_wdt_cnt (i.e., the maximum loop watchdog count).

**Figure 15:**
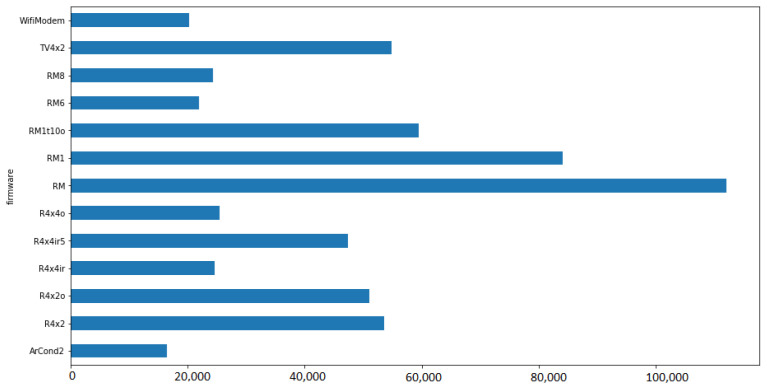
Readings count by firmware version.

The readings count by each of the 13 different firmware versions is presented in Figure 15.

**Figure 16:**
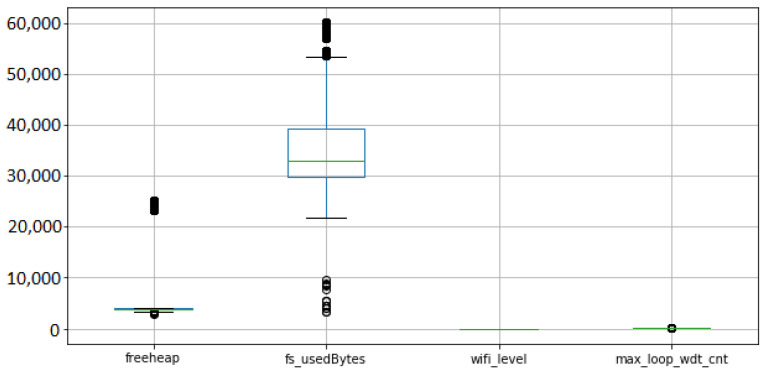
Summary of the four ESP8266 metrics.

A summary of the collected metrics of the 22 ESP8266 devices installed in the smart home is presented in the Figure 16.

**Figure 17:**
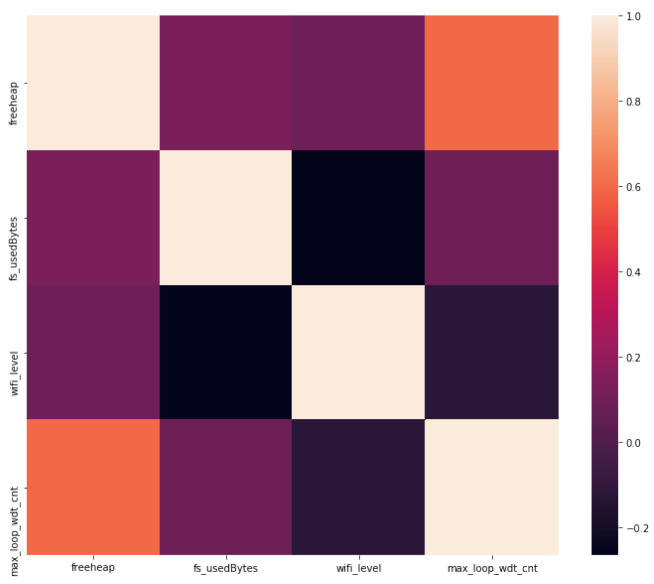
Smart home metrics correlation matrix.

The correlation matrix of the Figure 17 shows the negative correlation between wifi_level and freeheap, and between wifi_level and fs_usedbytes.

**Figure 18:**
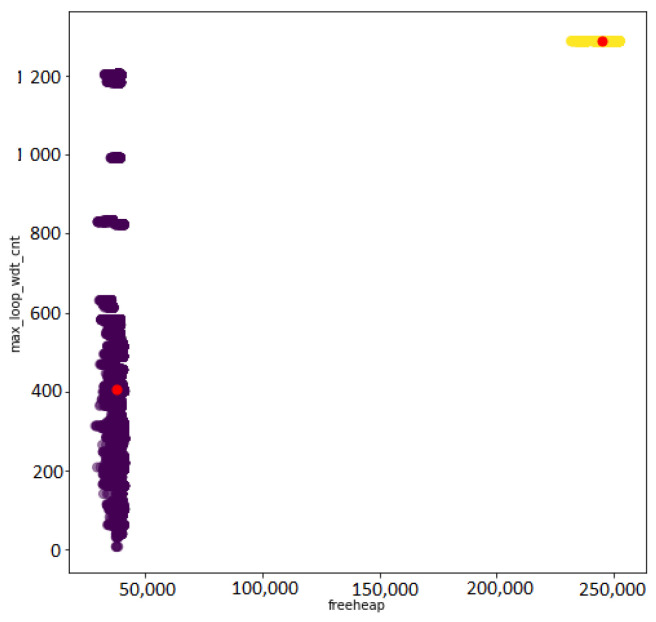
Smart home K-Means clusters.

The K-Means clustering algorithm was performed using the aforementioned four metrics with the total number of cluster of two corresponding to a good and bad status clusters. After the clustering, the cluster label is performed by the IoT developer based on the visual representation, such as the one represented in Figure 18.

The clustering results are summarized in Table 3. The firmware versions ArCond2, R4x2, RM6, RM8, and TV4x2 presented the worst status results, resulting from bad Wi-Fi signal and more integrated functionalities that may compromise performance. The firmware versions R4x2o, R4x4ir5, R4x4o, RM, RM1, RM1t10o, and WifiModem presented the best results, resulting from specialized functionality or better location for Wi-Fi level. The R4x4ir firmware version presented an average status.

With the trained model, it was possible to classify the IoT device under development status described in (1) in the good status cluster (i.e., cluster 1).

(2) $$\begin{array}{c} {}^{\prime }fs\_usedBytes^{\prime }:340858,\\ {}^{\prime }max\_loop\_wdt\_cnt^{\prime }:992,\\ {}^{\prime }freeheap^{\prime }:39112,\\ {}^{\prime }wifi\_level^{\prime }:-75 \end{array}$$

Considering the proposed framework, we describe the five steps considered:

1. Business drivers: the motivation is to scale the energy efficiency solution to smart homes in the same region, considering the different communication conditions;
2. Non-functional requirements: similar to the remote lab scenario, the performance efficiency requirement is prioritized, considering the resource utilization. Another relevant requirement is the maintainability, considering the testability characteristic.
3. Engineering view: the architectural solution was illustrated in the Figure 12, and described in detail.
4. Dynamic system evaluation: initial descriptions are described in Figure 13 and Figure 14. The historical clustering results are presented in Table 4, and one example of a status reading classified in the cluster 1 (good status according to the IoT developer rationale) is presented in (2).
5. Recommendations: considering that the deployed modules have the filesystem usage presented in Figure 16, it is desirable that the new IoT devices have filesystem usage of less than 300,000 bytes. Based on Table 4 obtained with the historical analysis of the 22 ESP8266 devices, from the 13 different firmware versions, the ArCond2, R4x2, RM6, RM8, and TV4x2 firmware versions must be monitored in detail by the Python Agent microservice. The devices with good status may have their monitoring cycle extended.

## 6. Discussion

The proposed framework and the TDD tool built with the K-Means algorithm could provide a rationale for the remote lab and smart home study cases, considering an end-to-end view. The RM-ODP standard was essential to provide a link between business drivers and non-functional requirements, and an approach to use the automated monitoring tool with the objective to analyze and assist in the IoT solution development process.

Some benefits found in the study cases: in the remote lab scenario the deployment and free memory conditions for devices with extended functionality were elucidated, and in the smart home scenario the status of 13 different firmware versions could be evaluated in an automated way, and provide insights for the monitoring frequency configuration.

The different conditions of the study cases show that our approach is not limited to specific scenarios or proprietary technologies. The study cases cover private and cloud computing environments in Brazil and USA, HiveMQ and Mosquitto MQTT brokers, SQL and No-SQL databases, and ESP8266 and ESP32 devices. The proof of concept was performed with these IoT development boards, but any board that supports the MQTT protocol might be integrated with the proposed TDD framework.

Another contribution is related to the applicability of the proposed method, considering that the proof of concept is open source for facilitated replication, and the communication is based on the open source standard MQTT [26]. The scripts for ESP32 and ESP8266 modules are, therefore, compatible with any cloud provider that implements the MQTT protocol, so our approach is compatible with any cloud back-end, minimizing the vendor lock-in problem. The PubSubClient library used for the device MQTT connection is also open source, unlike the Amazon Web Services (AWS) library only compatible with AWS back-end.

The validation of the proposed method with real devices might contribute to close the gap between proposed TDD methodologies and their effective application. We also expect that the proof of concept contributes as an example of using machine learning to generate automated tests for IoT.

However, a limitation of the proposed solution is that the automated tool must be trained in each different deployment scenarios for accurate assessment results. For example, in the remote lab the Internet connection could provide a remote clock sync in a faster way than in the smart home scenario, so an IoT device developed for the remote lab may not perform well in the smart home scenario.

Considering the most relevant related work found in the literature regarding IoT testing, [10] shows an overview of the challenges that arise when testing large IoT applications at the system level. They present a novel hybrid-simulation-based testing approach, and introduce various solutions to the challenges that arise when implementing this hybrid methodology. These challenges are mainly related to the IoT development pipeline, synchronization between real-life and the simulation environment and the scalability constraints of modern simulation techniques. The concept of hybrid simulation is to combine a complex behavior, represented by testbeds, with a more homogeneous behavior represented by simulation. Although it mentions that machine learning can be used, the article does not comment that it may have adopted such a solution.

The architecture proposed in [13] provides a high-level structural view of software, hardware, and communication components placed into layers for facilitating system design and development. However, it does not provide indications on where these components should be deployed. In order to provide a clear view of the different deployment locations for architectural components, they developed an approach of formalizing the highly distributed infrastructure of IoT systems and facilitating the creation of different deployment views for the mapping between layered architectural components into stage locations. In comparison with this work, our solution automated the implementation process and presented a better applicability of the proposed method, as the software and scripts are in the public domain and compatible with any provider that implements the MQTT protocol.

The MAPE-K framework (monitoring, analyze, plan, and execute with knowledge base), proposed in [16] requires detailed knowledge about contexts, sensors and actuators, and effects of the process in the real world for the modeling of objectives using the proposed methodology, requiring the use of more sophisticated approaches to formalize and deduce this knowledge in order to reduce the modeling effort. The work used BPM processes and systems that are more self-aware of their executions and self-adaptive through the feedback loop. However, applying feedback loops to ensure the result of process execution also introduces additional computational overhead, which can be counterproductive with respect to real-time constraints. The adopted solution requires additional resources for operation and communication, which may not be feasible in resource-constrained computing environments.

Researchers in [17] presented an alternative to improve the data transmission validation process. They implemented the test-driven development methodology (TDD) for the build out of validation application algorithms. As a result, they developed validation application that captures and analyzes data frames, in addition, compares the values of the monitored variable in each layer of the implemented model. Furthermore, the development reports the errors detected in the data transmission. This work has limitations, such as the high dependence on networks to monitor the performance of the designed application and also, the work did not perform testbeds to verify the approach in the wild with a real IoT implementation.

The Open-Multinet (OMN) framework [18] is based on ontologies to support the resource management in federated and distributed computing infrastructures. One of the known limitations of explicit knowledge-based systems is the laborious process of knowledge retrieval and update. Once the information is known, the queries may be acceptable to end-users, but its underlying update process may not be so dynamic. We could consider our proposed framework as a method to obtain resource information regarding specific scenarios in an automated way, thus providing more dynamism than the OMN framework.

Simulations and its underlying mathematical apparatus [19,20] are also relevant and may be used as an alternative to the proposed TDD approach with machine learning based on real testbed data.

To the best of the authors’ knowledge, it is the first TDD approach for IoT module automatic assessment which uses machine learning based on real testbed data.

## 7. Final Considerations

Considering the challenge of quality assessment in IoT systems, this work addressed the topic of automated non-functional requirements evaluation. The objective is to enhance the Testability non-functional requirement of IoT systems under development.

The proposal is evaluated in academic and smart home study cases with 25 devices, consisting of 15 different firmware versions collected in one week, with a total of over 550,000 IoT status readings. K-Means algorithm was applied to free memory available, internal temperature and Wi-Fi level metrics to automatically monitor the IoT devices under development to identify device constraints violation and provide insights for monitoring frequency configuration of different firmware versions. The clustering results enabled to assess if new data of a specific IoT device fits into bad or good status. In the remote lab, the bad status accounted for around 60% of the total readings.

The proposed framework guides the IoT system designer from the business domain to the non-functional requirement dynamic evaluation, based on RM-ODP [7] standard. The TDD tool is built with K-Means clustering algorithm application in real testbed data to acquire a high resemblance to real deployment scenarios. According to the proposed framework, the TDD tool could be used to identify IoT development problems earlier in the development process. The automated test results from the remote lab and smart home study cases indicate that the identification of device constraints violation is possible.

To the best of the authors’ knowledge, it is the first TDD approach for IoT module automatic assessment which uses machine learning based on real testbed data. Some of the related work carried out experiments in testbeds with real hardware devices but none of them used machine learning, which motivated the novel TDD approach validated with two study cases.

As future work, the comparison of K-Means with other unsupervised learning algorithms could be performed. Other possibility is to test different deployment conditions with synthetic firmware versions with known resource misuses to investigate how the number of clusters may affect the proposed automated tool performance. Supervised algorithms for time series prediction and firmware version classification tasks also might be applied to the collected data to expand the TDD approach to the security domain, focusing on anomaly detection.

A limitation of the proposed framework and the associated TDD tool based on machine learning is the need to know the total number of clusters beforehand, because the tool uses the K-Means algorithm. Such a limitation may be investigated in future work by testing other machine learning models. For example, graph-based deep learning approaches have been proposed as a promising solution for relevant problems (e.g., anomaly detection in communication networks [34,35], intrusion detection in IoT environments [36]). Additionally, the framework was validated with two case studies, therefore other scenarios may contribute to enhance its relevance.

## Figures and Tables

**Table 1:** Related works regarding IoT testing.

Reference	Publisher	Year	Simulation	Testbed	ML
Balador et al. [9]	MDPI	2018	no	yes	no
Bosmans et al. [10]	ACM	2019	yes	yes	no
Estebsari et al. [11]	MDPI	2019	yes	yes	no
YangQun Li [12]	MDPI	2018	yes	no	no
Zyrianoff et al. [13]	MDPI	2020	no	yes	no
Sivaselvan et al. [14]	IEEE	2020	no	yes	no
Farooq [15]	Oxford	2020	yes	yes	no
Seiger et al. [16]	Springer	2019	yes	yes	no
Fernández et al. [17]	UCC	2021	yes	no	no
Willner et al. [18]	MDPI	2017	no	yes	no
Kovtun et al. [19]	IEEE	2022	yes	no	no
Kovtun et al. [20]	Springer	2022	yes	no	no

**Table 2:** Remote lab ESP32 metrics summary.

Chipid	Freeheap			Internal_Temperature			Wifi_Level		
	Min	Mean	Max	Min	Mean	Max	Min	Mean	Max
5,861,220	215,592	220,818	222,332	50.0	50.4	52.2	−78.0	−70.8	−65.0
13,443,280	220,404	222,117	222,160	52.8	53.5	53.9	−83.0	−80.6	−79.0

**Table 3:** Remote lab ESP32 clustering results.

Firmware	Kmean	Count
arcond-ir	0—bad	224
	1—good	144
dht-tempumid	0—bad	498
	1—good	312

**Table 4:** Smart home ESP8266 clustering results.

Firmware	Kmean	Count
ArCond2	0—bad	16,384
R4x2	0—bad	53,644
R4x2o	1—good	51,049
R4x4ir	0—bad	11,242
	1—good	13,343
R4x4ir5	1—good	47,458
R4x4o	1—good	25,494
RM	1—good	112,168
RM1	1—good	84,060
RM1t10o	1—good	59,498
RM6	0—bad	21,886
RM8	0—bad	24,329
TV4x2	0—bad	54,758
	1—good	76
WifiModem	1—good	20,165

## Data Availability

Not applicable.

## References

[1] Cisco (2021). Cisco IoT Remote Operations. https://www.cisco.com/c/en/us/solutions/internet-of-things/remote-operations.html.

[2] O‘Hara D. (2021). Bridging the Divide: Getting IT and OT to Work Together for Industrial IoT—Cisco Blogs. Cisco Blogs.

[3] IBM (2021). Improving Systems Availability.

[4] Bauer M., Boussard M., Bui N., Carrez F., Jardak C., De Loof J., Magerkurth C., Meissner S., Nettsträter A., Olivereau A. Internet of Things–Architecture IoT-A Deliverable D1. 5–Final Architectural Reference Model for the IoT v3. 0. https://www.iot-a.eu/.

[5] Case J. (2011). Simple Network Management Protocol (SNMP). https://www.rfc-editor.org/rfc/rfc1098.

[6] (2021). System and Software Quality Models.

[7] Linington P.F. (1995). RM-ODP: The architecture. Open Distributed Processing.

[8] Beck K. (2002). Test-Driven Development by Example.

[9] Balador A., Kouba A., Cassioli D., Foukalas F., Severino R., Stepanova D., Agosta G., Xie J., Pomante L., Mongelli M. (2018). Wireless Communication Technologies for Safe Cooperative Cyber Physical Systems. Sensors.

[10] Bosmans S., Mercelis S., Denil J., Hellinckx P. (2019). Testing IoT systems using a hybrid simulation based testing approach. Computing.

[11] Estebsari A., Barbierato L., Bahmanyar A., Bottaccioli L., Macii E., Patti E. (2019). A SGAM-Based Test Platform to Develop a Scheme for Wide Area Measurement-Free Monitoring of Smart Grids under High PV Penetration. Energies.

[12] Li Y. (2018). An Integrated Platform for the Internet of Things Based on an Open Source Ecosystem. Future Internet.

[13] Zyrianoff I., Heideker A., Silva D., Kleinschmidt J., Soininen J.-P., Cinotti T., Kamienski C. (2020). Architecting and Deploying IoT Smart Applications: A Performance–Oriented Approach. Sensors.

[14] Sivaselvan N., Waqar A., Bhat V., Muttukrishnan R. Authentication and Capability-based Access Control: An Integrated Approach for IoT Environment. Proceedings of the 2020 12th International Conference on Communication Software and Networks (ICCSN).

[15] Farooq M.O. (2020). RIoT: A Routing Protocol for the Internet of Things. Comput. J..

[16] Seiger R., Huber S., Heisig P., Aßmann U. (2019). Toward a framework for self-adaptive workflows in cyber-physical systems. Softw. Syst. Model.

[17] Fernández L.F.P., Rosero J.P.R., González G.A.R. (2021). Multilayer Validation System for The Automation of Data in A Wsn Network with Iot Devices. Rev. Ing. Solidar..

[18] Willner A., Giatili M., Grosso P., Papagianni C., Morsey M., Baldin I. (2017). Using Semantic Web Technologies to Query and Manage Information within Federated Cyber-Infrastructures. Data.

[19] Kovtun V., Izonin I., Greguš M. (2022). Model of Information System Communication in Aggressive Cyberspace: Reliability, Functional Safety, Economics. IEEE Access.

[20] Kovtun V., Izonin I., Gregus M. (2022). The functional safety assessment of cyber-physical system operation process described by Markov chain. Sci. Rep..

[21] Sharma A., Maan A. (2022). Enabling Delay Tolerance in IoT based Vehicular Networks. Int. J. Sci. Res. Comput. Sci. Eng..

[22] Al-Fahdi J.A., Al-Yahyaai H.F., Al-Rashdi B.R., Muthu A. (2022). Smart Home Automation Based On Bluetooth with IR Receiver. Int. J. Sci. Res. Comput. Sci. Eng..

[23] Saemaldahr R., Thapa B., Maikoo K., Fernandez E.B. (2021). Reference Architectures for the IoT: A Survey. Computer Networks, Big Data and IoT.

[24] Gupta B.B., Quamara M. (2020). An overview of Internet of Things (IoT): Architectural Aspects, Challenges, and Protocols. Concurr. Comput. Pract. Exp..

[25] Bansal S., Kumar D. (2020). IoT Ecosystem: A Survey on Devices, Gateways, Operating Systems, Middleware and Communication. Int. J. Wirel. Inf. Netw..

[26] Banks A., Gupta R. (2021). MQTT Version 3.1.1. http://docs.oasis-open.org/mqtt/mqtt/v3.1.1/os/mqtt-v3.1.1-os.html.

[27] Arakaki R., Hayashi V.T., Ruggiero W.V. Available and Fault Tolerant IoT System: Applying Quality Engineering Method. Proceedings of the 2020 International Conference on Electrical, Communication, and Computer Engineering (ICECCE).

[28] Hayashi V.T., Arakaki R., Ruggiero W.V. (2020). OKIoT: Trade off analysis of smart speaker architecture on open knowledge IoT project. Internet Things.

[29] Newman S. (2015). Building Microservices: Designing Fine-Grained Systems.

[30] Pahl C., Jamshidi P. (2016). Microservices: A Systematic Mapping Study. https://bia.unibz.it/esploro/outputs/conferenceProceeding/Architectural-Patterns-for-Microservices-A-Systematic-Mapping-Study/991005773017601241.

[31] Beck K. (2001). Manifesto for Agile Software Development. https://agilemanifesto.org/.

[32] Kanungo T., DMount M., Netanyahu N.S., Piatko C., Silverman R., Wu A.Y. The analysis of a simple k-means clustering algorithm. Proceedings of the Sixteenth Annual Symposium on Computational Geometry.

[33] Nageswaran S., Arunkumar G., Bisht A.K., Mewada S., Kumar J.N.V.R.S., Jawarneh M., Asenso E. (2022). Lung Cancer Classification and Prediction Using Machine Learning and Image Processing. BioMed Res. Int..

[34] Jiang W. (2022). Graph-based deep learning for communication networks: A survey. Comput. Commun..

[35] Feng Y., Chen J., Liu Z., Lv H., Wang J. (2022). Full Graph Autoencoder for One-Class Group Anomaly Detection of IIoT System. IEEE Internet Things J..

[36] Zhou X., Liang W., Li W., Yan K., Shimizu S., Wang K.I.-K. (2022). Hierarchical Adversarial Attacks Against Graph-Neural-Network-Based IoT Network Intrusion Detection System. IEEE Internet Things J..

